# Papillary Thyroid Carcinoma in a Branchial Cleft Cyst: A Two-Case Single-Center Experience

**DOI:** 10.7759/cureus.93270

**Published:** 2025-09-26

**Authors:** Adriana Ferreira, Carla Quental, Teresa Lindo, Ana Lúcia Alves, José Guilherme Tralhao

**Affiliations:** 1 Surgery, Instituto Português de Oncologia de Coimbra Francisco Gentil, Coimbra, PRT; 2 Surgery, Unidade Local de Saúde de Coimbra, Coimbra, PRT; 3 Pathology, Unidade Local de Saúde de Coimbra, Coimbra, PRT

**Keywords:** branchial apparatus, branchial cleft cyst, cervical cystic lesions, papillary thyroid carcinoma, thyroid gland migration

## Abstract

Papillary thyroid carcinoma (PTC) arising within a branchial cleft cyst (BCC) is an exceptionally rare condition that presents diagnostic and therapeutic challenges. This study aims to describe two such cases identified in our institution and to review the relevant literature.

A retrospective review of the pathology database of Unidade Local de Saúde (ULS) de Coimbra in Coimbra, Portugal, from 2015 to the present identified two such cases. Clinical, pathological, and follow-up data were analyzed, and a brief literature review was performed.

The first patient, a 63-year-old man, presented with a right lateral cervical cystic mass that was surgically excised and found to be a papillary carcinoma. Subsequent total thyroidectomy revealed a microscopic focus of papillary carcinoma in the thyroid, and he received adjuvant radioactive iodine, remaining disease-free to date. The second patient, a 43-year-old man, underwent excision of a right lateral cervical BCC, with histology confirming papillary carcinoma arising within the cyst. Further evaluation demonstrated a synchronous 12 mm PTC with nodal involvement, treated with total thyroidectomy, neck dissection, and radioactive iodine, with no evidence of recurrence on follow-up.

These findings highlight the need to consider malignant transformation in BCCs and underscore the importance of thorough histopathological assessment of lateral neck masses.

## Introduction

The thyroid gland originates from the embryonic endoderm near the foramen cecum and migrates caudally to its final position anterior to the hyoid bone [1]. Ectopic thyroid tissue results from abnormal migration and may be found in locations such as the base of the tongue, larynx, trachea, esophagus, mediastinum, pericardium, diaphragm, or within a branchial cleft cyst (BCC) [2].

The branchial apparatus consists of six paired arches that form between the fourth and seventh gestational weeks. Normally, the branchial clefts and pouches are obliterated through mesenchymal invasion, completed by the eighth week. Failure of this process can result in persistent remnants, including BCCs, most commonly derived from the second arch. Second-arch anomalies arise because the second arch grows caudally and overlaps the third and fourth arches, creating an ectoderm-lined cervical sinus that normally involutes by the seventh week of gestation. Incomplete involution leaves epithelial remnants that may form a cyst, sinus, or fistula along the typical second-arch tract, from the anterior border of the sternocleidomastoid muscle, passing between the internal and external carotid arteries and terminating in the tonsillar fossa [2].

Papillary thyroid carcinoma (PTC) is the most frequent thyroid malignancy, accounting for 80% to 85% of cases. It typically exhibits indolent behavior, with a tendency for lymphatic rather than hematogenous spread. Cervical ultrasound and fine-needle aspiration (FNA) are the mainstays of diagnosis. Treatment strategies have evolved towards a more conservative approach, as reflected in the 2015 American Thyroid Association (ATA) Guidelines [3]. Depending on tumor size, extent, and risk factors, options range from lobectomy to total thyroidectomy, with or without lymph node dissection. In the absence of lymph node metastasis, prior neck irradiation, family history of thyroid carcinoma, or extrathyroidal extension, lobectomy alone may be considered for tumors up to 4 cm in diameter. Radioiodine ablation (RAI) is reserved for selected intermediate- and high-risk cases.

PTC arising within a BCC is an extremely rare entity, with few cases documented in the literature. Its diagnosis and management pose unique challenges. We report two additional cases of this uncommon presentation.

This report aims to characterize the institutional experience of Unidade Local de Saúde (ULS) de Coimbra regarding PTC arising within BCCs. By presenting two cases identified since 2015, we intend to contribute to the limited literature on this rare entity, highlight its clinicopathological features, and reinforce the importance of considering PTC in the differential diagnosis of lateral neck cystic masses, which includes a wide spectrum of congenital and acquired lesions such as thyroglossal duct cyst, dermoid or epidermoid cyst, thymic cyst, parathyroid cyst, laryngocele, and vascular malformations, as well as infectious or inflammatory lesions and cystic metastatic lymphadenopathy.

In collaboration with the Department of Pathology of ULS de Coimbra, a retrospective search was conducted to identify cases of PTC arising within a BCC. Due to limitations of the institutional pathology database, only cases from January 2015 to the present were accessible for review. Two cases were identified and are described in detail. Additionally, a brief review of the existing literature was performed to contextualize this rare presentation.

## Case presentation

Case 1

A 63-year-old man was seen in the surgery consultation at ULS Coimbra in 2020 because of a painless swelling in the right laterocervical region, which he had first noticed one month earlier. He reported a significant increase in size during the week prior to the appointment.

Investigation

A cervical CT scan revealed a cystic lesion located anterior to the sternocleidomastoid muscle, measuring approximately 51 × 30 mm in axial dimensions and 51 mm in longitudinal extension, suggestive of a second BCC. FNA cytology confirmed the diagnosis.

Treatment

**Figure 1:**
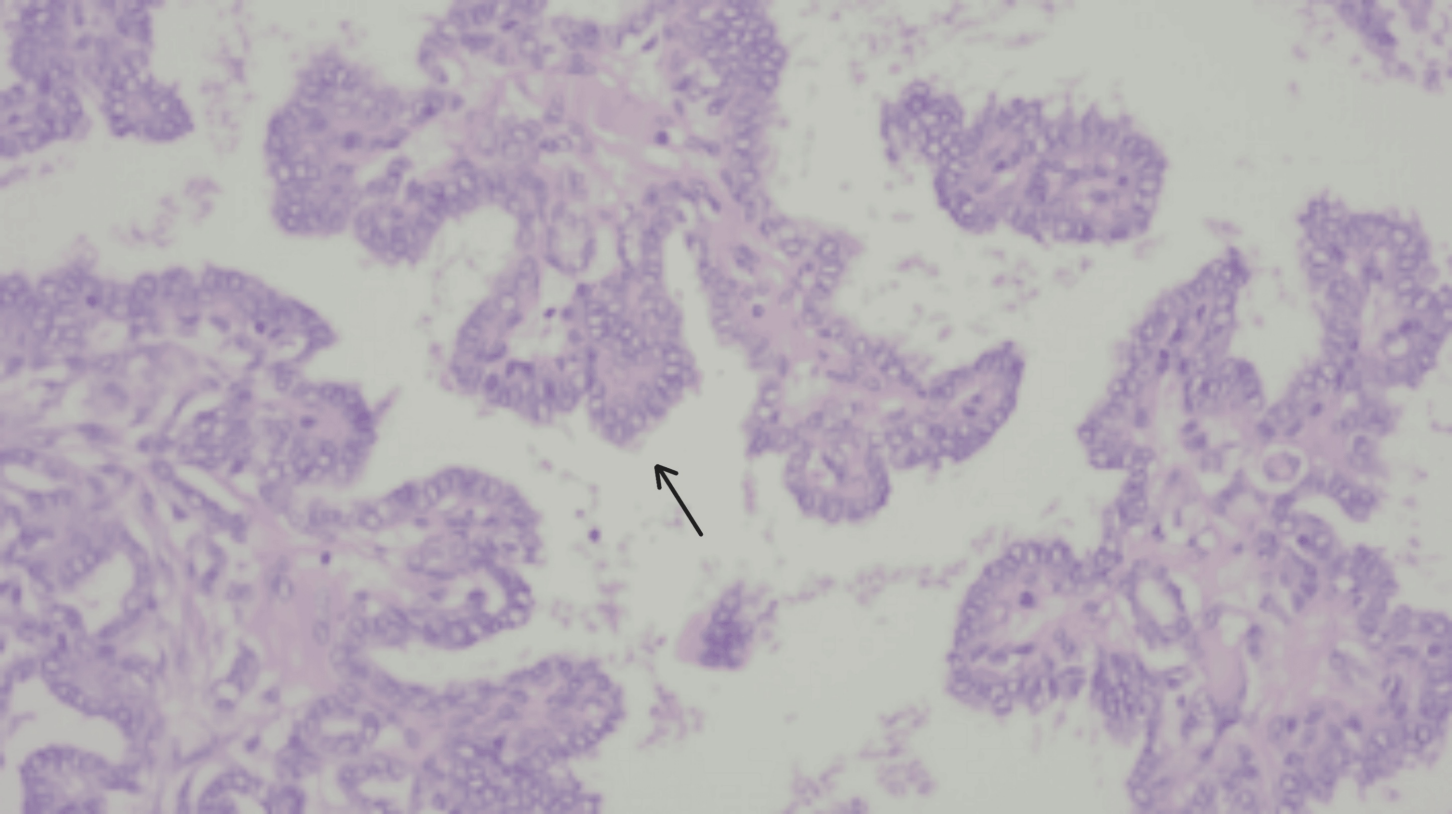
Intracystic papillary carcinoma (left), with papillary structures lined by cells with oval nuclei and chromatin margination; H&E, 100x (arrow).

The patient underwent surgical excision of the lesion under general anesthesia. Histopathological examination revealed a papillary carcinoma arising within a BCC, with intracystic growth and probable association with ectopic thyroid tissue. However, metastasis from a PTC could not be ruled out (Figure 1).

Two thyroid ultrasounds were performed six months apart, both with normal findings. Blood tests showed hypothyroidism, with normal values of thyroglobulin and anti-thyroglobulin antibodies (Table 1).

**Table 1:** Preoperative thyroid function tests and tumor markers in both patients.

Test	Patient 1	Patient 2	Reference Range
Thyroid-stimulating hormone (µIU/mL)	1.3	0.06	0.4-4.0
Free thyroxine (ng/dL)	0.7	1.1	0.7-1.5
Thyroglobulin (ng/mL)	23	<0.04	1.6 – 60
Anti-thyroglobulin antibodies (IU/mL)	0.7	0.5	<4

Levothyroxine 100 mcg was initiated.

**Figure 2:**
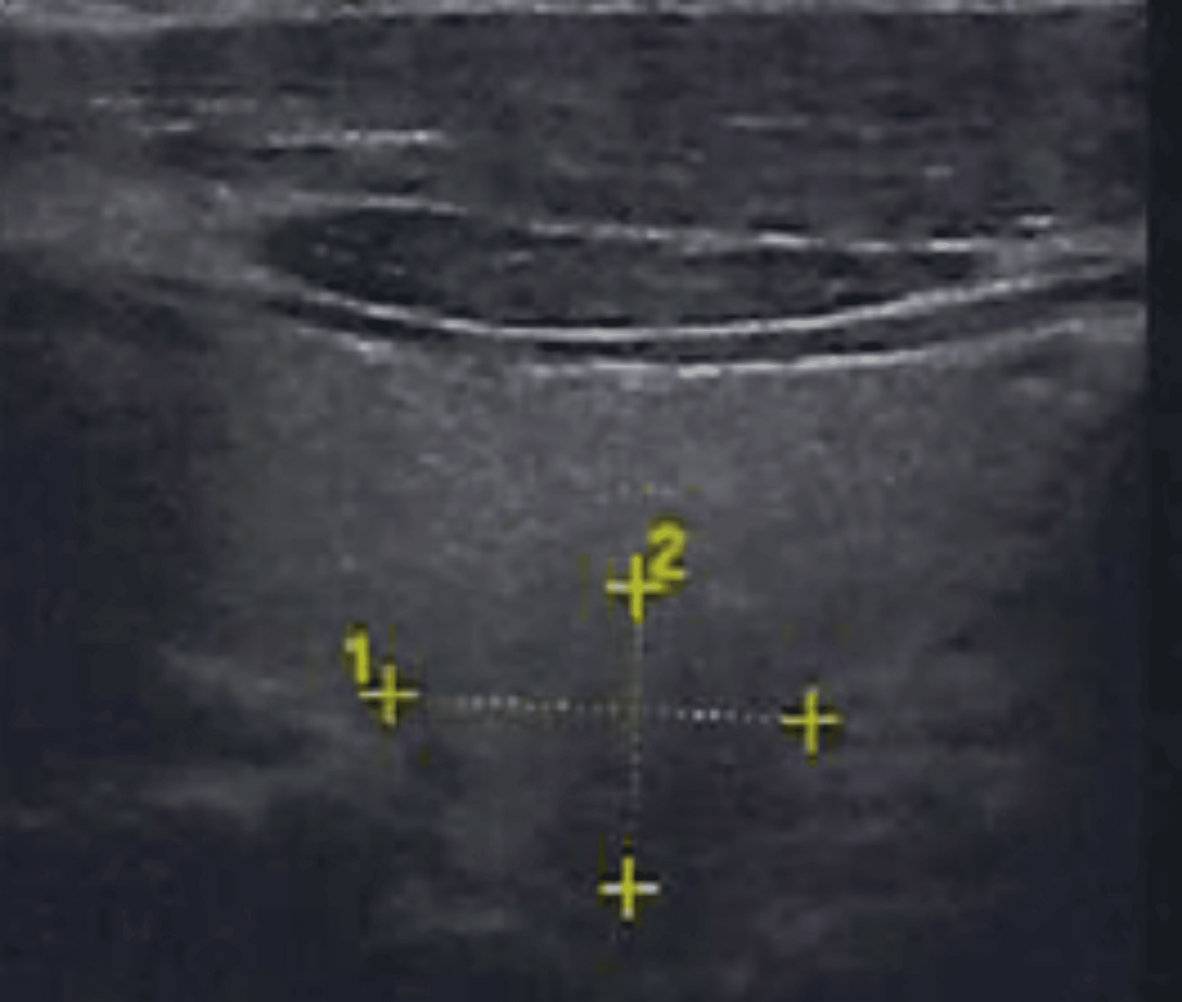
Neck ultrasound showing a solid nodular area in the right thyroid lobe; fine-needle aspiration (FNAB) classified it as Bethesda System Category II. The Bethesda System for Reporting Thyroid Cytopathology [4]

One year later, a third ultrasound identified a small, slightly hypoechoic and poorly defined nodule, measuring 7 × 5 mm, in the right lobe of the thyroid. FNA biopsy (FNAB) revealed a colloid/hyperplastic nodule (Figure 2; Bethesda System Category II [4]).

Despite the benign result found on the biopsy, and after discussion with the patient, a total thyroidectomy was performed. Although the thyroid nodule was cytologically benign (Bethesda System Category II), the decision for total thyroidectomy was made after multidisciplinary discussion and patient counseling. The rationale included the confirmed papillary carcinoma within the BCC, the possibility that this carcinoma could represent PTC arising in ectopic thyroid tissue, or, alternatively, metastatic disease from an occult thyroid primary that might not be detected by FNA. Total thyroidectomy was therefore recommended to allow definitive histologic assessment, enable radioactive iodine ablation, and facilitate long-term surveillance. Histological analysis of the surgical specimen revealed an incidental papillary microcarcinoma in the right lobe, measuring 0.9 mm, with complete excision (pT1aNxM0, American Joint Committee on Cancer (AJCC) 8^th^ edition tumor, node, metastasis (TNM) classification [5]). Posteriorly, the patient underwent RAI.

Follow-Up

The patient remained under regular surveillance by the surgery and endocrinology departments initially every six months and later annually. Follow-up included serial thyroglobulin and anti-thyroglobulin antibodies measurements, cervical ultrasonography, and whole-body scintigraphy, with no evidence of recurrence or distant metastases.

Case 2

Case Presentation

**Figure 3:**
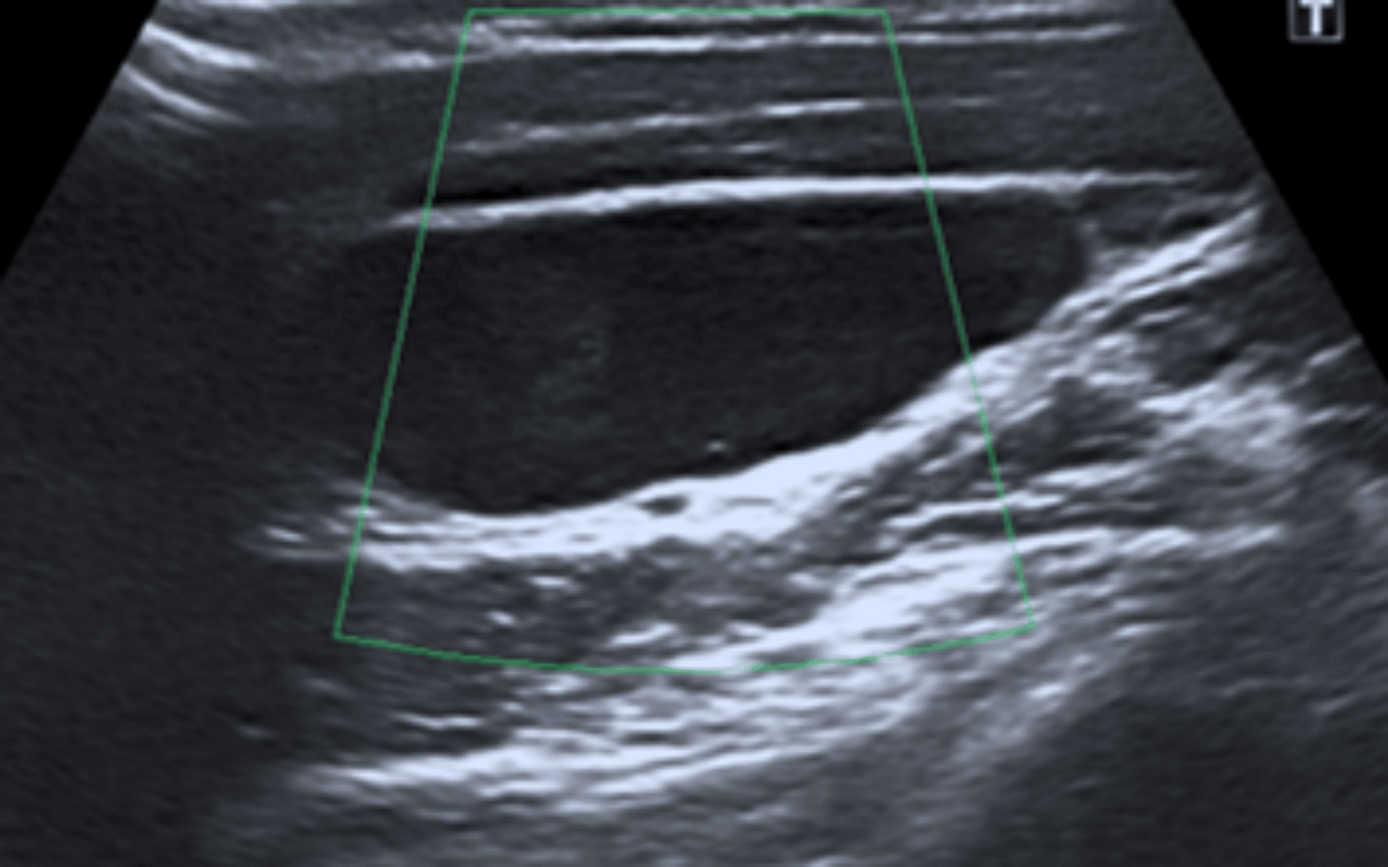
Ultrasound demonstrating a branchial cleft cyst.

A 43-year-old man was first seen in the surgery consultation at ULS Coimbra in 2018 due to a painless cervical mass (Figure 3).

Investigation

**Figure 4:**
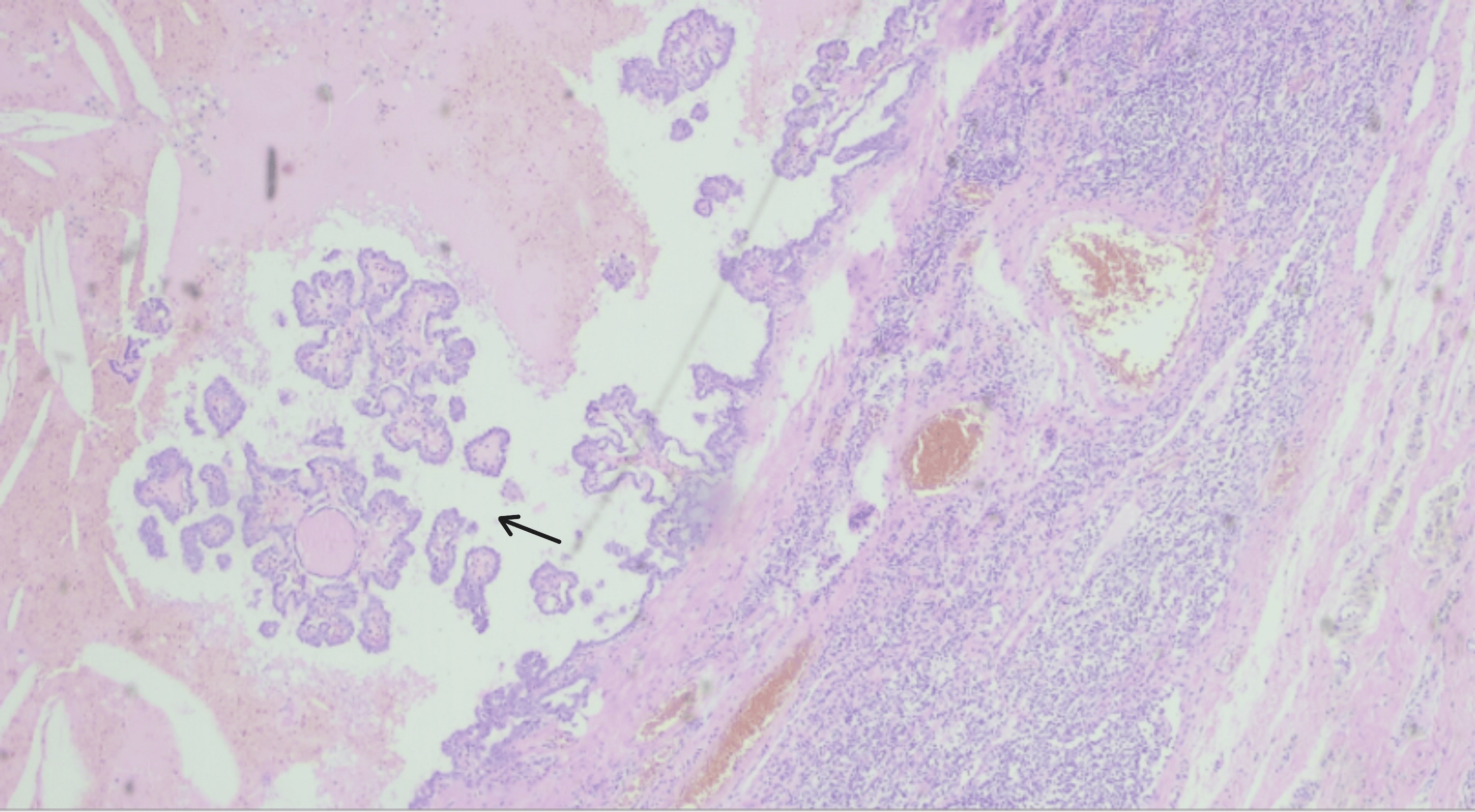
Papillary carcinoma within a cyst, with possible association with ectopic thyroid tissue or metastasis from the papillary thyroid carcinoma; H&E, 40x (arrow).

He underwent multiple cytologies and biopsies, all of which were inconclusive. Surgical excision of the cervical lesion was performed. Histopathological analysis revealed a papillary carcinoma arising in a right cervical cystic lesion, but it was not possible to differentiate between a carcinoma arising in a BCC and a metastasis from a primary thyroid tumor (Figure 4).

A cervical CT scan revealed a small nodule in the posteroinferior aspect of the right thyroid lobe and a right laterocervical adenopathic conglomerate (level III). FNA of the thyroid nodule was classified as a follicular lesion of undetermined significance (FLUS).

Treatment

The patient underwent total thyroidectomy with modified radical neck dissection (levels II-VI on the right). Histopathological examination revealed a classic variant of PTC (12 mm) in the right lobe, extending into perithyroidal fat, with metastases in 7 of 24 excised lymph nodes (pT3N1bMx, AJCC 8th edition TNM classification [5]). Postoperatively, he was treated with radioactive iodine (I-131) under endogenous stimulation.

Follow-Up

He remained under oncology follow-up every six months. No malignant recurrence or distant metastases were confirmed. Serial surveillance with thyroglobulin and anti-thyroglobulin antibody measurements and cervical ultrasound remained unremarkable.

## Discussion

An initial literature search identified 15 published cases of PTC occurring in a BCC. A subsequent PubMed search using the keywords “Papillary thyroid carcinoma” AND “Branchial cleft cyst” revealed 12 additional cases. Including the two cases reported in this article, a total of 29 cases have now been documented in the literature, as summarized in Table 2.

**Table 2:** Reported cases of papillary thyroid carcinoma in a branchial cleft cyst F: female; M: male

No	Author/Year	Age (years)/Gender	Treatment	Pathological results	Reference
1	Sidhu et al. (2000)	42/F	Surgical excision of the branchial cyst and total thyroidectomy	No evidence of carcinoma within the thyroid gland	[6]
2	Balasubramaniam et al. (1992)	34/F	Surgical excision of the branchial cyst	Thyroidectomy was not performed	[7]
3	Matsumoto et al. (1999)	46/F	Surgical excision of the branchial cyst and hemithyroidectomy	Adenomatous goiter, no evidence of carcinoma within the thyroid gland	[8]
4	Cappellani et al. (2004)	29/F	Surgical excision of the branchial cyst and total thyroidectomy	Papillary thyroid carcinoma within a branchial cleft cyst; no evidence of carcinoma within the thyroid gland	[9]
5	Fumarola et al. (2006)	36/F	Surgical excision of the branchial cyst and total thyroidectomy	Multifocal papillary carcinoma	[10]
6	Mehmood et al.(2006)	31/M	Surgical excision of the branchial cyst and total thyroidectomy	No evidence of carcinoma within the thyroid gland	[11]
7	Lanzafame et al. (2006)	29/F	Surgical excision of the branchial cyst and total thyroidectomy	No evidence of carcinoma within the thyroid gland	[12]
8	Chi et al. (2009)	24/M (The other case is a cystic change of a metastatic lymph node from thyroid papillary carcinoma)	Surgical excision of the branchial cyst and total thyroidectomy	Branchial cleft cyst with concurrent lymph node metastasis from thyroid papillary carcinoma	[13]
9	Park et al. (2010)	49/M	Surgical excision of the branchial cyst, selective right neck dissection, and total thyroidectomy	No evidence of carcinoma within the thyroid gland	[14]
10	Cho et al. (2011)	41/F	Right lateral neck dissection and total thyroidectomy	Nodular hyperplasia of the right lobe without evidence of carcinoma within the thyroid gland	[15]
11	Kuhwaha et al. (2012)	34/F	Surgical excision of the branchial cyst, right modified radical neck dissection, and total thyroidectomy	No evidence of carcinoma within the thyroid gland	[16]
12	Gollahalli et al. (2013)	35/M	Surgical excision of the branchial cyst and total thyroidectomy	Thyroid papillary carcinoma arising in ectopic thyroid tissue within a branchial cyst; no occult primary tumor of the thyroid was found.	[17]
13	Karras et al. (2013)	35/F	Surgical excision of the branchial cyst and total thyroidectomy	No evidence of carcinoma within the thyroid gland.	[18]
14	Ruhl et al. (2013)	20/M	Surgical excision of the branchial cyst and total thyroidectomy and selective neck dissection (central and ipsilateral)	Thyroid papillary carcinoma within the branchial cleft cyst; no evidence of carcinoma within the thyroid gland.	[19]
15	Sagit et al. (2013)	41/F	Surgical excision of the branchial cyst; the patient refused thyroidectomy	Thyroidectomy was not performed	[20]
16	Tazegul et al. (2018)	22/M	Surgical excision of the branchial cyst; total thyroidectomy	Papillary thyroid carcinoma within the branchial cleft cyst; thyroidectomy revealed two foci of micropapillary thyroid carcinoma; four out of 14 lymph nodes were positive for metastasis.	[21]
17	Gür et al. (2019)	35/M	Surgical excision of the branchial cyst, total thyroidectomy, and left functional neck dissection. Radioactive iodine therapy.	Papillary thyroid carcinoma within the branchial cleft cyst. Two focal papillary thyroid carcinomas of 1.5 cm and 1 cm were identified in the left lobe of the thyroid gland, and the larger showed invasion into the thyroid gland capsule. One PTC focus of 1.2 cm was identified in the right lobe. In addition to the metastasis into the branchial cleft cyst, five metastatic lymph nodes were observed in the neck dissection material.	[22]
18	Hong et al. (2019)	42/M 29/M 43/M	Surgical excision of the branchial cyst for all three cases	No thyroidectomy; total thyroidectomy showed no malignancy; total thyroidectomy showed no malignancy	[23]
19	Cooc et al. (2020)	49/F	Right neck mass excision, lymph node dissection, total thyroidectomy, and neck exploration with otolaryngology.	Branchial cleft cyst with involvement by papillary thyroid carcinoma; invasive papillary thyroid carcinoma; metastatic papillary thyroid carcinoma in several right level II through IV lymph nodes	[24]
20	Tsung (2021)	55/M	Total thyroidectomy and radial neck dissection	Occult follicular variant of thyroid carcinoma	[1]
21	Marotta et al. (2021)	28/M	Surgical excision of the branchial cyst and total thyroidectomy	Classic papillary carcinoma occurring in a branchial cleft cyst; three separate foci of well-differentiated papillary thyroid carcinoma in the right lobe, with a small micro-carcinoma focus within the left lobe. Enlarged lymphadenopathy discovered in level 6 of the neck was identified as a site of metastases with extension outside of the lymph node into the soft tissue. Other foci were contained within the surgical margins.	[25]
22	Wang et al. (2024)	48/M	Surgical excision of the branchial cyst; hemithyroidectomy and functional neck lymph node dissection	Coexistence of a branchial cleft cyst with metastatic papillary thyroid carcinoma, as well as papillary carcinoma in the right thyroid lobe. Lymph node metastasis was observed in the central and levels III of the right neck.	[26]
23	Khawaja et a. (2024)	20/F	Surgical excision of the branchial cyst; total thyroidectomy and selective neck dissection	Classic papillary carcinoma occurring in a branchial cleft cyst. No evidence of carcinoma within the thyroid gland.	[27]
24	Atram et al. (2024)	24/F	Surgical excision of the branchial cyst and total thyroidectomy	Papillary carcinoma in a branchial cleft cyst; papillary microcarcinoma in the thyroid.	[28]
25	Skalias et al. (2024)	46/M	Surgical excision of the branchial cyst; Total thyroidectomy with selective neck dissection including levels III, IV, and VI	Papillary carcinoma in a branchial cleft cyst; papillary thyroid carcinoma with no lymph node metastasis.	[29]
26	Vroegindewey et al.(2025)	53/M	Surgical excision of the branchial cyst; Total thyroidectomy	Papillary carcinoma in a branchial cleft cyst; papillary thyroid carcinoma	[30]
27	Present cases	63/M , 43/M	Surgical excision of branchial cyst and total thyroidectomy for patient 1, and surgical excision of branchial cyst and total thyroidectomy with modified cervical lymph node dissection on the right side of levels II, III, IV, V, and VI for patient 2	Papillary carcinoma occurring in a branchial cleft cyst; papillary microcarcinoma in the right lobe of the thyroid. Papillary carcinoma in a right cervical cystic lesion, not being possible to differentiate between papillary carcinoma in a branchial cleft cyst and a metastatic lesion of a primary thyroid tumor; a classic variation of papillary carcinoma measuring 12 mm located in the right lobe of the thyroid, extending to the perithyroidal adipose tissue and tangential to the surgical margin, with metastases in seven of the 24 excised nodes, two of which belong to the central compartment (T3N1bMx)	

The differential diagnosis of a painless lateral neck mass is broad, encompassing congenital, inflammatory, and neoplastic entities such as thyroglossal duct cyst, cystic hygroma, dermoid or epidermoid cyst, thymic or parathyroid cyst, laryngocele, vascular malformations, infectious abscesses, and cystic metastatic lymphadenopathy. Although exceedingly rare, PTC arising within a BCC should also be considered among these possibilities.

Sidhu et al. proposed diagnostic criteria for primary PTC arising in a BCC, which include the presence of epithelial lining or subepithelial lymphoid tissue, normal thyroid tissue adjacent to the carcinoma within the cyst wall, and no evidence of PTC in the thyroid gland or elsewhere [6].

In both of our cases, occult PTC in the thyroid gland was ultimately identified. This underscores a key diagnostic challenge, determining whether carcinoma within a BCC represents metastatic spread from an undetected primary thyroid tumor or a synchronous primary tumor arising within the cyst itself.

As supported by the small number of reported cases, PTC arising in a BCC is an exceedingly rare entity. Reporting additional well-documented cases is essential to enrich the limited body of evidence and to improve recognition of this unusual presentation. Multicenter collaborations and larger prospective studies are needed to clarify the true incidence, refine diagnostic criteria, and define optimal therapeutic strategies and long-term follow-up protocols.

## Conclusions

In conclusion, although lateral cystic neck masses may arise from diverse causes, clinicians should remain alert to the possibility of PTC developing within a BCC. The differential diagnosis of painless lateral neck lesions is broad and should include this rare entity among other congenital, inflammatory, and neoplastic conditions. Distinguishing a true primary carcinoma in a BCC from metastatic disease originating in the thyroid remains a diagnostic challenge, particularly when a synchronous thyroid lesion is present.

By documenting these two additional cases, we contribute to the limited body of literature and highlight the need for multicenter studies to better define incidence, refine diagnostic criteria, and establish optimal therapeutic strategies and follow-up protocols. This report emphasizes the exceedingly rare occurrence of PTC within a BCC and the importance of careful clinicopathological correlation to avoid misdiagnosis.
